# Multilayer block copolymer meshes by orthogonal self-assembly

**DOI:** 10.1038/ncomms10518

**Published:** 2016-01-22

**Authors:** Amir Tavakkoli K. G., Samuel M. Nicaise, Karim R. Gadelrab, Alfredo Alexander-Katz, Caroline A. Ross, Karl K. Berggren

**Affiliations:** 1 Department of Electrical Engineering and Computer Science, Massachusetts Institute of Technology, 77 Massachusetts Avenue, Cambridge, Massachusetts 02139, USA; 2 Department of Materials Science and Engineering, Massachusetts Institute of Technology, 77 Massachusetts Avenue, Cambridge, Massachusetts 02139, USA

## Abstract

Continued scaling-down of lithographic-pattern feature sizes has brought templated self-assembly of block copolymers (BCPs) into the forefront of nanofabrication research. Technologies now exist that facilitate significant control over otherwise unorganized assembly of BCP microdomains to form both long-range and locally complex monolayer patterns. In contrast, the extension of this control into multilayers or 3D structures of BCP microdomains remains limited, despite the possible technological applications in next-generation devices. Here, we develop and analyse an orthogonal self-assembly method in which multiple layers of distinct-molecular-weight BCPs naturally produce nanomesh structures of cylindrical microdomains without requiring layer-by-layer alignment or high-resolution lithographic templating. The mechanisms for orthogonal self-assembly are investigated with both experiment and simulation, and we determine that the control over height and chemical preference of templates are critical process parameters. The method is employed to produce nanomeshes with the shapes of circles and Y-intersections, and is extended to produce three layers of orthogonally oriented cylinders.

## Abstract (web summary)

Block copolymer self-assembly can successfully generate monolayers of complex nanopatterns. Here, the authors orthogonally self-assemble 2 or 3 layers of distinct molecular weight block copolymers, and fabricate complex nanomesh structures without alignment or high-resolution lithographic templating.

## Graphical abstract

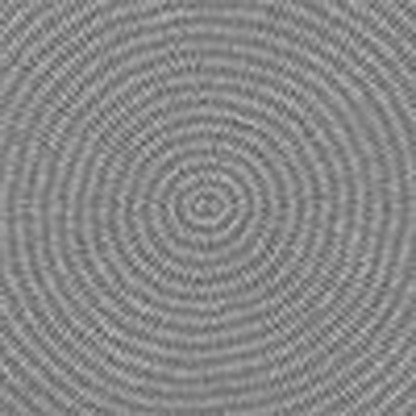

Monolayers of block copolymer (BCP) microdomains made by templated self-assembly have been highly successful at producing both simple and complex two-dimensional (2D) patterns, for example, bends and junctions, as well as different morphologies on a single substrate^1^,^2^,^3^,^4^,^5^,^6^,^7^,^8^,^9^,^10^,^11^,^12^. Beyond 2D, reports have investigated BCP self-assembly in three dimensions (3D), such as in thin films and nanopores^13^,^14^,^15^,^16^,^17^,^18^. One of the most interesting and applicable 3D BCP structures is the nanomesh formed by bilayer or multilayer stacks of orthogonal line patterns. Nanomeshes can be useful in several technologies including photonic materials^19^,^20^, graphene nanomesh devices^21^,^22^ or next-generation integrated-circuit architecture^23^. In addition, nanomeshes could be used for fabricating bit-patterned media where orthogonal arrays of rectangular features as defined by nanomesh structures, are required^24^.

Previous methods for nanomesh fabrication have shown advantageous versatility and functionalities^25^,^26^,^27^,^28^,^29^,^30^,^31^. A particular stacking method, the sequential transfer printing of layers^25^,^26^,^30^, was based on precise orientational alignment of the layers, and required multiple process steps including release of the pattern from the transfer substrate and separate templating steps for each layer. Furthermore, this method was limited to fabrication of uniform, large-area meshes and did not produce locally aperiodic patterns such as bends, junctions or circular shapes. Alternatively, a single-step method^31^ was demonstrated to fabricate bilayer meshes in only one annealing step. This approach required a dense electron-beam lithography (EBL) template and the templating features left a residual trace within the final mesh pattern^32^. In addition, only square arrays of rectangular holes were fabricated, without any circular constructions. A method of fabricating mesh patterns without requiring stacking or high-resolution templates would enable greater patterning speed and flexibility.

We introduce here a self-assembly method for the fabrication of multilayer nanomesh patterns in which grating patterns from the previous self-assembly step were used as a template to self assemble a subsequent orthogonal BCP grating layer with a different period. This method includes the key features of facilitating self-assembly of two or more layers of different molecular weight BCPs orthogonal to the underlying BCP templating layer; fabricating circular and rectangular nanomesh structures in complex arrangements; and patterning without high-resolution lithography or transfer printing.

Previous reports have shown that BCPs may be templated to align orthogonal to the direction of the underlying topographically modulated substrate or lithographic pattern^9^,^33^,^34^,^35^,^36^,^37^,^38^,^39^,^40^,^41^,^42^,^43^,^44^,^45^. Hong *et al*. have shown that in-plane BCP cylinders can align in-plane orthogonal to the ridges of a faceted sapphire and silicon substrate depending on the topography of the ridges and the BCP film thickness^41^,^42^. Sapphire substrates allowed in-plane alignment orthogonal to the ridges for both thick and thin BCP layers but silicon substrates produced orthogonal alignment for only thick films. The cause of this difference was explained by the shape of the ridges obtained from silicon versus sapphire facets. Park *et al*. reported that lamellar microdomains of BCPs could be templated to align orthogonal to ridges of lithographically patterned substrates^35^,^37^,^38^,^39^. Prior reports of BCP-on-BCP alignment are limited to in-plane parallel alignment of cylinders or lamellae registered to out-of-plane cylinders^16^,^46^,^47^,^48^, and there is still room for the understanding of the conditions required to produce orthogonal self-assembled BCP features.

Here, we develop an in-depth understanding of the orthogonal self-assembly mechanism through experimental and simulation-based results. With this understanding, we show the capability of the orthogonal self-assembly method to align three BCP layers in plane perpendicular to each other, laying the foundation for extension of the method to many layers. The fundamental mechanism was demonstrated with both BCP and EBL patterns as bottom layer templates, thus locally controlling bilayers of mesh in bends, junctions and circles, and including sub-10-nm feature sizes. In addition to local pattern control, the orthogonal self-assembly effect was shown to persist over large substrate areas.

## Results

### Fabrication of multilayer nanomesh

**Figure 1:**
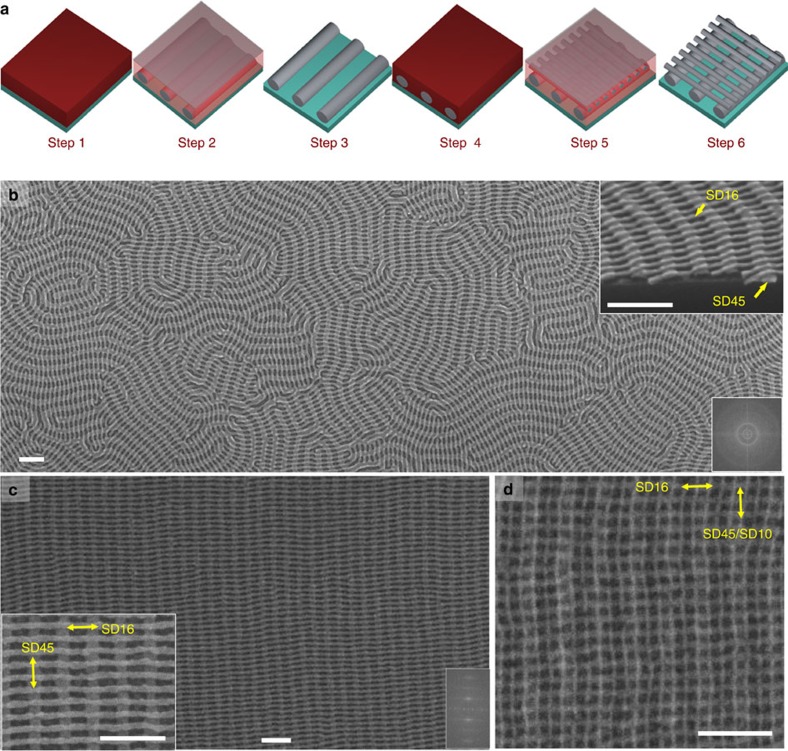
Nanomesh patterns via the orthogonal self-assembly method. (**a**) The major steps of the fabrication process. Step 1, spin-coating a BCP film on a Si substrate; step 2, BCP annealing to promote microphase separation forming a monolayer of in-plane PDMS cylinders; step 3, reactive ion etch to remove the polystyrene matrix of the BCP and leave a monolayer of ox-PDMS cylinders on the substrate; step 4, spin-coating a second monolayer of BCP on the ox-PDMS microdomains; step 5, BCP annealing of the second layer; step 6, reactive ion etch to remove the polystyrene matrix of the BCP and leave a mesh-shaped ox-PDMS bilayer pattern on the substrate. (**b**) SEM image of a complex nanomesh in which the bottom layer, on bare Si, has a short correlation length. The top inset is a cross-sectional SEM image of the bilayer mesh. The bottom inset is the 2D Fourier transform with distinct circles representing the periodic nature of the mesh without long-range order. (**c**) SEM image of well-ordered nanomesh patterns in which the bottom layer of cylinders, on PDMS-brushed Si without a template, has a long correlation length. The left inset is a zoomed-in SEM image of two-layer nanomesh pattern. The right inset is the 2D Fourier transform. The twofold, repeating symmetry is represented by the peaks along the *x-* and *y*-axes. (**d**) SEM image of a three-layer nanomesh pattern in which the first and third layers of cylinders (SD45 and SD10) are parallel to each other and orthogonal to the middle layer of cylinders (SD16). Scale bars, 100 nm.

Figure 1a shows the major steps of the orthogonal self-assembly method. The first layer of the BCP was assembled on the substrate and then used as a template to orthogonally align the following layer. Three different in-plane cylindrical-morphology poly(styrene-*b*-dimethylsiloxane) (PS-*b*-PDMS) BCPs were used in the experiments: 45.5 kg mol^−1^ (SD45; fraction of PDMS *f*_PDMS_=32%, period *L*_0_=43 nm), 16 kg mol^−1^ (SD16; *f*_PDMS_=31%, *L*_0_=19 nm) and 10.7 kg mol^−1^ (SD10; *f*_PDMS_=25%, *L*_0_=15 nm). In the first step, a monolayer of PS-*b*-PDMS BCP was spin coated on square silicon substrates of 1.5 cm^2^ size (either grafted with a PDMS or PS brush, or prime, as received from the manufacturer, see Methods for details). After the BCP spin-coating, solvent annealing was used to promote microphase separation and form in-plane PDMS cylindrical microdomains within a PS matrix. Toluene vapour was used for SD45 BCP solvent annealing, and acetone vapour for SD16 and SD10 BCP annealing^31^,^49^. The film thickness to achieve a monolayer of in-plane cylindrical PDMS microdomains was dependent on the chemical functionality and topographic pattern on the silicon substrates. After solvent annealing, a CF_4_ reactive ion etch (RIE; 5 s for SD45, 3 s for SD16 and 2 s for SD10) was used to remove the PDMS air-surface layer, followed by an O_2_ RIE (22 s for SD45, 14 s for SD16 and 12 s for SD10) to remove the PS matrix and leave a monolayer of oxidized-PDMS (ox-PDMS) collapsed cylinders on the substrates with a height of less than about half the cylinder diameter. In the next step, a monolayer of another PS-*b*-PDMS BCP was spin coated on top of the BCP templating pattern (without any further substrate functionalization). The bottom layer pattern had already been converted to insoluble ox-PDMS^28^ because of O_2_ RIE, and therefore was not removed by the solvent during the second spin coating nor deformed during the subsequent solvent annealing. Then as described above, solvent annealing was used to promote microphase separation in the second layer. During the solvent annealing, the top layer of PDMS cylinders oriented orthogonally to the bottom ox-PDMS cylinders. The two-step RIE process was used to remove the PS matrix and leave a bilayer ox-PDMS mesh-shaped structure on the surface of the substrate. To pattern a third layer of mesh, we repeated the BCP film deposition, annealing and etching a third time. To verify the results, we repeated the two-layer mesh on more than 200 samples and the three-layer mesh on more than 10 samples.

Figure 1b–d shows selected nanomeshes from this process. In Fig. 1b, SD16 BCP ox-PDMS cylinders were oriented orthogonal to SD45 ox-PDMS cylinders. The bottom layer SD45 was spin coated on a bare silicon substrate and therefore had a short correlation length (the distance over which the orientation of microdomains is maintained^50^). In this image, SD16 cylinders were orthogonal to the bottom fingerprint patterns of SD45 despite the extensive defectivity and curvature in the SD45 cylinders. The top inset shows a cross-sectional scanning electron microscope (SEM) image of the mesh-shaped structure. The observed sagging of the ox-PDMS cylinders in the inset is thought to be a result of the oxygen plasma etching. Once the supporting PS block is removed with oxygen plasma, the cylinders collapse onto the underlying template and substrate. Similar results were observed in a previous report^31^ and characterized with transmission electron microscope tomography^51^.

**Figure 2:**
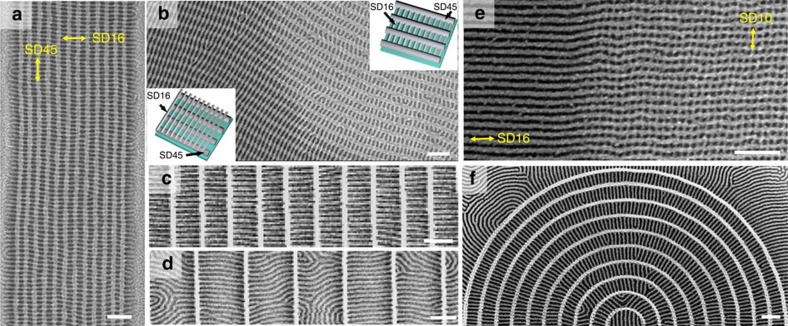
SEM images of orthogonal self-assembly of BCP on top of BCP or HSQ patterns. (**a**) Mesh-shaped structure in an interference-lithography trench template. The SD45 BCP cylinders (bottom layer) are oriented in the *y*-direction along the trenches and SD16 BCP cylinders (top layer) were orthogonally self-assembled along the *x* direction (see Supplementary Fig. 1 for larger image and GISAXS results). (**b**) Nanomesh with graded thickness of top-layer BCP (see Supplementary Fig. 3 for larger image). (**c**,**d**) Orthogonal self-assembly of SD16 BCP over HSQ line arrays with 100 and 200 nm periods (see Supplementary Fig. 4 for other periods). (**e**) Ultra-high-density nanomesh patterns of low-molecular-weight BCP bilayers (see Supplementary Fig. 5 for larger image). (**f**) Orthogonal self-assembly of SD16 BCP over concentric EBL-patterned circles (see Supplementary Fig. 6 for full-size image and different period of templating circles). Scale bars, 100 nm.

Figure 1c shows SD16 BCP on top of SD45 BCP with a larger correlation length, resulting in an ordered ortholinear array of rectangular nanoholes. The improved order of the SD45 cylinders in the bottom layer was accomplished by increased annealing time and by functionalizing the bare silicon substrate with a PDMS brush, enhancing the chain mobility during the annealing process^52^. The left inset in Fig. 1c shows a close-up SEM image. The in-plane dimensions of the mesh-shaped holes are 9 × 21 nm^2^, that is, an aspect ratio of more than 2. In both of the SEM images in Fig. 1b,c, the SD16 cylinders crossed over the top of SD45 cylinders. By decreasing the thickness of the top layer (SD16) to less than a monolayer of microdomains, the SD16 cylinders spanned between the bottom SD45 cylinders instead of crossing over the top. This is shown in Fig. 2b where there is a gradient in the SD16 film thickness, such that the cylinders on the right show less continuity across the SD45 microdomains.

The orthogonal self-assembly method potentially provides a mesh-shaped structure over a full wafer without the need for high-resolution lithography or multiple templating and alignment processes, via an overall bottom-layer orientation that can be guided with a single lithography step. Figure 2a shows a sample in which the SD16 was templated by SD45, which itself was guided over the entire sample by a silica trench template fabricated by interference lithography. The grazing-incidence small-angle X-ray scattering (GISAXS) results in Supplementary Fig. 1 confirm that the mesh-shaped pattern was globally aligned over a substrate area of a few square millimetres.

The orthogonal self-assembly method can be extended to more than two layers. Figure 1d shows the results with three BCP layers, each orthogonal to the one below it. SD45, SD16 and SD10 BCPs formed the bottom, middle and top layers, respectively. SD10 and SD45 gratings are parallel to each other, and orthogonal to the middle SD16 grating. Supplementary Fig. 2 shows another combination of these three layers. The periodicity of the top layer cylinders was measured and determined to be similar to the *L*_0_ of an untemplated monolayer film of the same BCP, and therefore the underlying gratings do not seem to perturb the top layer period.

### Main parameters for achieving nanomesh

We now show, using both experimentation and simulation, the effect of template parameters on orthogonal alignment, specifically the chemical functionalization, template periodicity and template height.

**Figure 3:**
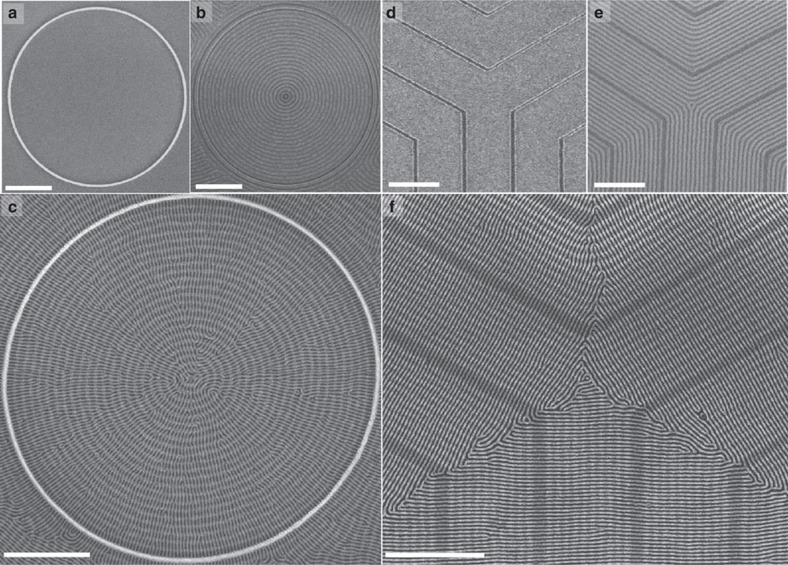
SEM images of lithographic and nanomesh patterns in circular and Y-junction templates. (**a**–**c**) Circular template. (**a**) The HSQ EBL template. (**b**) The SD45 BCP cylinders are templated by the PDMS-functionalized HSQ circles to form concentric rings. (**c**) Circular nanomesh pattern from SD16 over SD45. (**d**–**f**) Y-junction template. (**d**) The sacrificial PMMA EBL template. (**e**) The SD45 BCP cylinders are templated by the PMMA pattern to form three-direction arrays of cylinders with a central intersection. The PMMA template was removed during the etching process. (**f**) A Y-junction nanomesh pattern from SD16 over SD45. Scale bars, 500 nm.

Nanomeshes were formed regardless of whether the Si substrate was initially attractive to PDMS due to PDMS-brush grafting (Figs 1c and 3c), to PS due to PS-brush grafting (Supplementary Fig. 7 and Fig. 3f) or as-received (Fig. 1b). In contrast, chemical modification of the bottom layer ox-PDMS pattern was critically important. We found that orthogonal alignment of the second layer was promoted when the bottom BCP pattern was not functionalized after it had been etched. The understanding of this requirement was clarified through self-consistent field theory (SCFT) modelling, as discussed later, which corroborated the experimental results. The importance of this requirement is consistent with previous reports^1^,^16^ in which orthogonal assembly was not produced when the bottom layer template was attractive to either block due to a brush layer. In these cases, the cylinders in the top BCP layer oriented either parallel to or with no clear correlation to the underlying BCP pattern^16^.

We then studied the effect of the bottom pattern periodicity on orthogonal self-assembly. The SEMs in Supplementary Fig. 4 show that mesh-shaped structures appeared over a wide range of bottom-layer periodicities, larger than the *L*_0_ of the top layer BCP. Orthogonal self-assembly occurred on line-array templates with a range of periodicities, 30–100 nm (templates were fabricated using EBL from hydrogen silsesquioxane (HSQ) resist at an as-spun thickness of 50 nm). The orthogonal alignment started to degrade for template periodicities larger than 100 nm (see Fig. 2c,d). Although the ordering was lost for large periodicities, both blocks (PS and PDMS) still wetted the HSQ walls, indicating a non-preferential wetting condition (see Fig. 2d). The largest pitch at which orthogonal self-assembly is preserved is believed to be kinetically limited and may be improved by increasing the diffusivity or annealing time. Orthogonal self-assembly was also observed when the period of the bottom templating layer was smaller than the equilibrium period, *L*_0_, of the top-layer BCP. As shown in Fig. 2e, perpendicular orientation of SD16 BCP, with *L*_0_=19 nm, was templated by SD10 BCP, with a smaller *L*_0_ of 15 nm. The resulting bilayer mesh (Fig. 2e) also offered a method for fabricating a rectangular array of high-resolution dense nanoholes (7 × 9 nm^2^), which could be useful for high-areal-density applications. Figure 2f and Supplementary Fig. 6 show nanomeshes with concentric circle templates and orthogonally oriented SD16 BCP ‘spoke' patterns for two of the wide range of periodicities (30–100 nm). These images highlight that even if the in-plane cylinders of the bottom layer are curved, we can observe orthogonal self-assembly, similar to the linear results in Supplementary Fig. 4. As an extension, the orthogonal self-assembly is observed independent of the orientational order and periodicity of the bottom layer (Fig. 1b and Supplementary Fig. 8).

Our study showed the height of the bottom layer pattern is extremely important in producing orthogonal orientation. Supplementary Fig. 9 shows three examples in which bottom layers (HSQ templates and BCP patterns) were too thin to induce orthogonal orientation of the top layer. As the height decreased below 10 nm (for an SD16 top layer), the orthogonal orientation degraded. SCFT modelling confirmed this dependence on feature height, which will be described below in detail. Image analysis of nanomesh patterns on a variety of line-array templates with different pitches and heights was used to extract the orientational angle of top-layer SD16 lines, and the fitted distributions are plotted in Supplementary Fig. 10. The deviation of cylinders away from 0° (the orthogonal direction) was higher for thinner templates as compared with thicker templates. The sharpest peak was observed for SD16 cylinders on an SD45 template.

To produce aperiodic patterns, various EBL-fabricated templates were used to direct the self-assembly of the bottom BCP layer. Figure 3a shows an HSQ circle template and Fig. 3b the concentric circles of SD45 produced by templated assembly on the PDMS-brushed HSQ circle. Figure 3c shows SD16 on top of the concentric circles of SD45. The SD16 formed a radial pattern, which accommodated the change in circumference of the SD45 circles by creating line defects. As a comparison, the SD16 in Fig. 2f, where the bottom template was concentric HSQ circles, created fewer defects in order to accommodate changes in circumference because the segments of cylinders in each ring had little or no connection to each other.

Figure 3f shows locally controlled mesh-shaped structures in a Y-shape geometry with an intersection in the middle. In Fig. 3d, a sacrificial Y-junction template was fabricated with negative-tone poly(methyl methacrylate) (PMMA) EBL resist^32^. The cross-linked PMMA patterns were functionalized with a PS brush (unlike the HSQ patterns) and SD45 was assembled within them, shown in Fig. 3e after RIE. The etching of the SD45 also led to removal of the PMMA lines, leaving ox-PDMS cylinders parallel to the arms of the Y and with bends and a junction in the middle of the template. The top SD16 BCP layer formed cylinders orthogonal to the SD45 (Fig. 3f), bridging the gaps where the PMMA template had been present, and thus forming three large ‘grains' with 120° boundaries. Supplementary Fig. 7 shows another example of mesh-shaped structures in three different in-plane directions.

### SCFT simulation results

**Figure 4:**
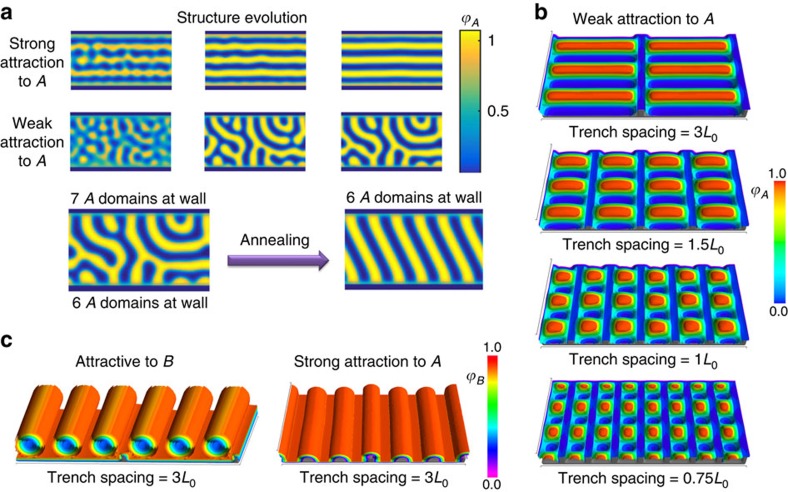
SCFT simulation of the self-assembly of a diBCP under confinement. (**a**) 2D model shows structural evolution under strong and weak surface attraction to polymer *A* (*f*_A_=0.5, *χN*=17). Strong attraction to polymer *A* creates a uniform wetting layer of *A* that forces the rest of the domains to lie parallel to walls. Weak attraction allows microdomains to emanate from surfaces towards the bulk. Trapped states result in concentric arches with large radii but annealing recovers the orthogonal lamellar structure. (**b**) 3D model (*f*_A_=0.27, *χN*=30) equilibrium isosurfaces of *A* (minority) density for weakly attractive topography showing orthogonal structure between trenches, independent of trench spacing. (**c**) Parallel isosurfaces of *B* density resulted when topography is attractive to *B* (majority) or strongly attractive to *A*. Top wetting layer in 3D simulations is removed to reveal structure. Computational cell is doubled to demonstrate trench periodicity. Dark grey substrate is added for clarity.

Our experimental findings motivated an investigation using SCFT simulation to understand the parameters that dictate the orientation of cylindrical microdomains on a patterned surface. In the first instance, a 2D simulation of an *A-B* BCP between two parallel walls (a simplification representing the experimental bottom-layer BCP pattern) was conducted. The volume fraction of block *A* (*f*_A_) was 0.5 and the calculations used a *χN* value of 17, where *χ* is the Flory–Huggins interaction parameter and *N* is the degree of polymerization. The equal volume fraction of the two blocks caused the BCP to form striped domains that represent a 2D projection of in-plane cylinders (or out-of-plane lamellae). A masking method^53^,^54^ was employed to apply confinement between sidewalls that were attractive to block *A*. Figure 4a shows the development of the microdomains as the simulation evolved^55^. It is noted that the structural evolution in SCFT does not represent a kinetic pathway to achieve the final morphology. Instead, SCFT seeks minima in the energy landscape that are physically realizable. Hence, we extracted structural information from the metastable states on how the BCP self assembles under confinement with different surface wetting conditions. Strong attraction at the walls created a uniform wetting layer that covered both surfaces, which enforced order parallel to the walls and created a propagating front that aligns the microdomains, resulting in an in-plane parallel structure^56^.

On the contrary, weak attraction at the walls caused a significantly different structure evolution. An interconnected network of microdomains was formed in which microdomains protruded orthogonal to the surface into the bulk. Such an orthogonal alignment was suppressed by the strong wall attraction in the previous case. Clearly, for a perfectly orthogonal structure to form, the protruding microdomains on both surfaces must bridge the gap between walls. SCFT structural evolution on the other hand showed that such bridging could be prevented by trapped metastable states such as loops with both ends on the same wall. This observation reproduced remarkably well the experimental work shown in Fig. 2d. In fact, these loops could have a large radius of curvature and could be mistakenly characterized as a structure parallel to the walls (see Supplementary Fig. 9b). Nonetheless, seemingly parallel structures had both ends normal to the confining sidewalls, and extensive annealing of this metastable structure allowed an orthogonal structure to form (see Fig. 4a). The gain in free energy on forming the orthogonal structure was 0.044 *nVk*_B_*T*, where *n* is number of chains, *V* is the volume accessible to the polymer and *k*_B_*T* is the thermal energy. It is important to note that in both metastable and annealed structures, the number of polymer domain pairs at the walls was almost identical as indicated on Fig. 4a. SCFT simulation further asserted that there was a range of wall attraction strength in which an orthogonal structure will still be generated independent of wall separation distance^54^,^57^. In this range, an orthogonal structure could be stabilized by a negative line tension at the walls^53^,^58^. The *AB* interface could intersect the wall at an angle and broaden the energetically favourable polymer *A* coverage to the wall, which lowers the free energy of the orthogonal structure^58^.

The transition from orthogonal to parallel orientation with increased surface attraction strength is shown in the supplementary for an exactly commensurate wall separation (that is, the wall separation is an integer multiple of *L*_0_, the BCP equilibrium spacing. See images in Supplementary Fig. 11). This transition shows that the trench walls do not have to be strictly neutral to obtain orthogonal alignment, and provides an explanation for the experimental work in which orthogonal templating of cylindrical microdomains was produced for HSQ or ox-PDMS templates, both of which might be expected to exhibit at least a weak preferentiality to PDMS. Finally, it is noted that the description of wall attraction as strong or weak is qualitative, although it serves to distinguish two distinct regimes of polymer self-assembly.

A 3D simulation is required to capture details such as the height and separation of the confining walls, the shape of the minority microdomains and their junction with the topographic features. 3D SCFT simulations of a confined *A-B* BCP were carried out with *χN*=30 and *f*_A_=0.27. The film thickness was kept constant at *L*_0_ with the top (air) surface preferential to *A*, as expected from the low surface energy of PDMS^45^,^59^.

Among the model parameters, the chemical functionality of the topographical template was particularly important in determining the shape of the microphase-separated structure. Figure 4b shows results produced when the topography was weakly attractive to *A.* Block *A* formed discontinuous flattened cylinders extending orthogonal to the trench walls, as seen experimentally for thin BCP films and following the results of the 2D model. Supplementary Fig. 12 shows bridging of *A* microdomains across the trench, as observed experimentally in Supplementary Figs 4,6 and 8. The size of the neck grew to produce a uniform cylinder at equilibrium. The orthogonal cylindrical structure prevailed independent of trench width. Such orientation cannot be justified by incommensurability between *L*_0_ and the trench width as the orthogonal structure was observed even for commensurate cases at trench widths of *L*_0_ and 3*L*_0_ (see Fig. 4b).

Modelling was also performed at a higher surface attraction strength to *A*, analogous to a template functionalized by a PDMS-brush^16^. The microdomains in this case oriented parallel to the trench walls, and the cylinders adhered to the trench walls as shown in Fig. 4c and in Supplementary Fig. 13. The morphology depended on the wall height: for shallow trenches that were attractive to *A*, microdomains from adjacent trenches combined to create extended cylinders crossing multiple trenches (Supplementary Fig. 14), but barrier crossing was suppressed when the wall height exceeded ∼10% of the film thickness. Although these results generally follow what was observed experimentally, the height of the experimental topography needed to influence the self-assembly of the upper BCP layer was greater than that in the model. This may be a result of BCP film swelling during solvent annealing, which was neglected in our simulations. We also determined that it is the surface properties of the walls rather than the contour of the walls that defined the orthogonal orientation, although previous reports suggest that more investigation of this characteristic could prove promising^60^.

When the substrate was attractive to the majority *B* block (Fig. 4c), the substrate was covered with a layer of *B* and the cylinders of *A* were raised above the surface. The cylinders aligned parallel to the trench walls in the range of strength of attraction investigated in this study. In addition, there was interplay between trench width and barrier height, particularly at incommensurate trench spacings. As the computational cell width was ∼3*L*_0_, three cylinders per computational cell were forming for shallow trenches independent of the number of trenches in the cell. The cylinders formed without regard to the presence of the mesas (see Supplementary Fig. 15). On the contrary, when the wall is taller the cylinders formed within the trenches with significant deformation in their cross-sectional shape (squeezed cylinders if the trench was too narrow to accommodate them, or flattened ellipses if the trench was too wide, as shown in Supplementary Fig. 16). The results of the simulation hint at a universal behaviour that can be harnessed to create meshed patterns, parallel patterns or density multiplication by controlling the preferentiality and the template height to direct each consecutive self-assembled layer.

## Discussion

In summary, the central result of this work is to show how nanoscale 3D crosspoint structures can be synthesized by orthogonal self-assembly of multilayers of BCPs with different molecular weights. We fabricated circular and rectangular nanomesh structures with non-trivial features including bends and junctions with global and local directions defined by lower-resolution patterning in an easily implemented process. We demonstrated orthogonal self-assembly of BCPs on both BCP structures and patterns written by EBL, as well as substrate-wide alignment using interference lithography patterns. SCFT simulations provided an understanding of the mechanism of the orthogonal self-assembly method, and the key importance of the template functionalization and height. Orthogonal self-assembly is suitable for fabrication of dense arrays of rectangular holes with aspect ratio (length/width) determined by the period of the two BCPs, and can be extended to make three or potentially more layers, creating multilayer nanomesh structures. The robustness of the method suggests its applicability to other BCP systems and to making functional 3D structures by multiple assembly and pattern transfer steps, accomplished, for example, by backfilling^13^, sequential infiltration synthesis^61^ or damascene processes^62^,^63^.

## Methods

### Characteristics of the BCPs

Three different poly(styrene-*b*-dimethylsiloxane) (PS-*b*-PDMS) BCPs were purchased (Polymer Source, Inc.) and prepared for these experiments. A 45.5 kg mol^−1^ weight polymer (SD45; *f*_PDMS_=32%, PDI (poly dispersity index) ∼1.09) was dissolved 2 wt% in propylene glycol monomethyl ether acetate for spin coating. A 16 kg mol^−1^ weight polymer (SD16; *f*_PDMS_=31%, PDI∼1.08) was dissolved 0.7 wt% in cyclohexane for spin coating. A 10.7 kg mol^−1^ weight polymer (SD10; *f*_PDMS_=25%, PDI∼1.14) was dissolved 0.7 wt% in cyclohexane for spin coating. The thickness of the film required to make the first monolayer of cylindrical microdomains was dependent on the functionality of the silicon substrates. A greater film thickness was required to form a monolayer of cylinders for PDMS-functionalized substrates compared with PS-functionalized substrates because of the formation of a PDMS wetting layer at the surface. Thickness was determined by spinning speed. The range of monolayer thickness and the periodicity *L*_0_ of BCPs for a monolayer with different functionality of the substrate were, respectively, 29–35 nm and 43 nm for SD45, 22–28 nm and 19 nm for SD16 and 22–28 nm and 15 nm for SD10. Thickness was measured by spectral reflectometry (Filmetrics).

### Application of BCPs and polymer brushes

Dissolved BCPs were spin coated on square silicon substrates of 1.5-cm^2^ size to a thickness which resulted in a monolayer of in-plane cylinders. To apply a PDMS homopolymer brush, a 1 wt% solution of 0.8 kg mol^−1^ hydroxyl-terminated PDMS (Polymer Source, Inc.) in toluene was spin coated at 3,000 r.p.m. for 30 s. For a PS brush, a 1 wt% solution of 1.2 kg mol^−1^ hydroxyl-terminated PS (Polymer Source, Inc.) in propylene glycol monomethyl ether acetate was spin coated at 3,000 r.p.m. for 30 s. Substrates were thermally annealed at 170 °C for 15 h in a vacuum oven (∼20 torr), then submerged in a bath of room-temperature toluene for 15 min, and rinsed with toluene to remove any ungrafted brush polymer.

### Solvent annealing of the BCP thin films

Solvent annealing was carried out with samples on a stage inside a beaker filled with a small volume of the annealing solvent liquid and thus solvent vapour. The process, as previously discussed^31^,^49^, is briefly described below.

SD45 was annealed in a capped glass beaker filled with 2 ml of toluene for 5–6 h, and then immediately quenched in ambient air to freeze-in the annealed structure. SD16 and SD10 BCPs were annealed in a beaker filled with 1 ml of acetone for about 6 h until all of the acetone evaporated. The beaker measured 5 cm in diameter and 1.5 cm in height. The Petri dish lid measured 10 cm in diameter and was almost flat but allowed slow evaporation of the solvent. The SD45 samples were placed on glass slides inside the beaker at a height of 0.6 cm, and the SD16 and SD10 samples at a height of 0.8 cm.

### Reactive ion etching

RIE was used to remove the PDMS top-coat and PS matrix of the BCP monolayer to leave behind oxidized, in-plane PDMS cylinders. For SD45 BCP, 5 s of CF_4_ (50 W, 15 mTorr) was used to remove the layer of PDMS that forms at the top of the BCP film, and 22 s of O_2_ (90 W, 6 mTorr) was used to remove the PS matrix. For SD16 BCP, 3 s of CF_4_ and 14 s of O_2_ were used. For SD10, 2 s of CF_4_ and 12 s O_2_ were used.

### Template fabrication

For samples in which the BCP was templated by electron-beam-lithographic structures, HSQ and PMMA resists were used.

### HSQ patterning

HSQ was purchased from Dow Corning (XR-1541, 1 and 4% solids in methyl isobutyl ketone). Templating patterns were written on a Raith 150 EBL tool, at an accelerating voltage of 30 kV, working distance of 6 mm and current of 250–300 pA. Line patterns were written at a wide array of doses, from 13,785 to 705,640 pC cm^−1^, depending on the pattern density. The samples were subsequently developed in a salty aqueous solution of 4% NaCl and 1% NaOH^64^ for 4 min at room temperature and then rinsed in deionized water for 2 min to remove residual salt and blown dry with N_2_ gas. Developed patterns were cleaned and fully oxidized by oxygen plasma ashing (50 W, 0.35 Torr) for 2 min.

HSQ patterning was used for template fabrication because it is a common resist material used in previous directed self-assembly of BCPs^1^,^32^ and experimentally exhibits similar chemical preferentiality as oxidized PDMS. Although we do not suggest that HSQ is a replacement for oxidized PDMS in the presented work, we took advantage of the facile engineerability of EBL to vary the experimental parameter space. In this regard, HSQ, as a well-reported resist used for BCP DSA, was chosen to mimic and test the orthogonal self-assembly method.

### PMMA patterning

PMMA (950 PMMA, 950 kg mol^−1^ in anisole) was purchased from MicroChem Corp and diluted 1:8 in anisole. Thin films were prepared by spin-coating at 4,000 r.p.m. for 60 s, followed by a post-bake at 200 °C for 2 min to remove residual solvent. PMMA thickness on samples was measured to be about 40 nm by spectral reflectometry. Templates were fabricated using EBL exposure of the PMMA as a negative-tone resist. PMMA is typically a positive-tone resist but it behaves as a negative-tone resist at high doses (6–10 times higher than HSQ). This change in tone happens due to a carbonization process^65^. Samples were subsequently developed by sonication in acetone for 8 min, rinsed with isopropyl alcohol for 1 min, and blown dry with N_2_.

### Large-area orientation analysis with GISAXS

GISAXS was performed at Argonne National Laboratory (Lemont, IL, USA) to confirm mesh-pattern self-assembly over large areas. GISAXS has been previously reported for the inspection of large areas of BCP nanopatterns^9^,^66^. Trenches were fabricated in oxidized silica via interference lithography over the entire 12 × 12 mm^2^ sample^67^. The trenches had a pitch of 1 μm, height of 35 nm and trench width of about 600 nm. SD45 BCP was spin coated to form a monolayer of cylinders within the trench and solvent annealed as previously described. After RIE, arrays of oxidized PDMS cylinders, aligned with the trench length, were left on the substrate. On top of this bottom-layer template, a SD16 BCP solution was spin coated to form a monolayer on top of the SD45 cylinders and solvent annealed. After RIE, a mesh pattern remained within the trenches, covering the substrate. An SEM image of the pattern is shown in Supplementary Fig. 1.

The mesh-patterned template sample was analysed at beamline 8-ID-E at Argonne National Laboratory using X-rays with energy 7.35 keV. Samples were measured under ambient conditions at multiple incident angles (ai: 0.1–0.3°) around the measured critical angle of 0.20° and the reported images are from an incident angle 0.20°. The sample-to-detector distance was 2,154 mm and the exposure time was roughly 1 s. The width of the probing beam was 100–200 μm, wide enough to inspect multiple trenches simultaneously.

In the top right image of Supplementary Fig. 1, the sample was aligned such that the beam direction was parallel to the SD45 cylinders and trenches. The multiple peaks along **q**_y_ indicate long-range order of the SD45 cylinders and the real-space pitch of the peaks (38–42 nm) is consistent with the BCP pitch as measured by top-down SEM (43 nm) The deviation can possibly be a result of domain pitch distortion within trench templates^67^. Multiple fainter angled grating truncation rods can be observed at lower **q**_y_-angles, likely representative of tails of the diffraction from the trench grating.

In the bottom right image of Supplementary Fig. 1, the sample was rotated 90° and aligned with the SD16 cylinders parallel to the beam direction. The peak at roughly **q**_y_=0.03 indicates order of the SD16 cylinders and the real-space pitch of the peak (∼18 nm) is consistent with the BCP pitch as measured by top-down SEM (19 nm). Very faint peaks can be observed at higher **q**_y_ values, indicating some long-range order, although much of this order was disrupted by the trenches and defects. There are peaks at **q**_y_=∼0.01, likely from diffraction off the beam stop.

### SEM imaging

Imaging of the samples was performed on either a Raith 150 EBL system with a LEO column or a Zeiss Merlin SEM. The Raith 150 microscopy was performed at 10 keV accelerating energy, 6 mm working distance, with an aperture of 30 μm for a current of around 150 pA. The Zeiss Merlin microscopy was performed at 5–10 keV accelerating voltage, ∼4 mm working distance and 100–150 pA. For cross-sectional imaging, microscopy was performed on the Zeiss Merlin similar to above, but at an angle tilt of 80°.

### Film thickness measurement for BCP and EBL resist

BCP and EBL resist film thicknesses were measured via a Filmetrics F20 spectral reflectometer. Inspection wavelengths were 250–1,500 nm.

### Image analysis for determination of orientation density probability

The angle of the top-layer SD16 cylinders with respect to the normal of the bottom-layer templating lines was determined through image analysis of scanning-electron micrographs. The specifically analysed bottom-layer templates included HSQ lines at pitches of 40, 60 and 80 nm, both thin and thick HSQ line heights, and SD45 cylinder lines. Matlab (Mathworks, version R2015a) was used to process the micrographs and extract the orientations. The programme first corrected the image contrast and the greyscale image was converted to a black-and-white image, at a specific intensity threshold, of only the templating features. This image was subtracted from the original image, thus leaving only the orthogonally aligned SD16 cylinders. This greyscale image was converted to black and white at the necessary intensity threshold and processed with morphological operations, thus identifying each SD16 cylinder as an independent object. The angles of the objects were determined by finding the angle of the major-axis of a representative ellipsoid estimation. An angle of 0° was specified for an orthogonal orientation. These angles were accumulated for each image, and the standard deviation was determined for plotting of the normal-distribution fit.

### SCFT simulations

SCFT simulation is a powerful approach to capture the complex physics governing the self-assembly of BCP. Here, we briefly describe the formalism of SCFT simulations of diBCPs.

We considered a monodispersed melt of *n A*-*B* BCP of volume *V,* with each diblock molecule composed of *N* segments. The *A* and *B* blocks consists of *fN* and (*1-f*)*N* chain segments, respectively. The interaction between the dissimilar blocks was controlled by a Flory–Huggins parameter, *χ*. Within the mean-field approximation, the free energy of the system *F* is expressed in terms of field variables


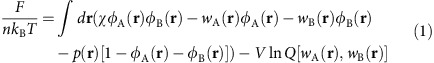


where *φ*_α_(**r**) is the volume fraction of species *α* at position **r**. *Q*[*w*_A_*,w*_B_] is the partition function of a non-interacting single polymer chain in external fields *w*_α_(**r**). The polymer was assumed to be incompressible, so the constraint *φ*_A_(**r**)*+ φ*_B_(**r**)*=*1 was enforced through a pressure field *p*(**r**). The free energy *F* was compared with the thermal energy *k*_B_*T*.

The single chain partition function can be evaluated as follows


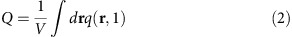


where *q*(**r***, s*) is a restricted chain partition function (propagator) that could be calculated by solving a modified diffusion equation


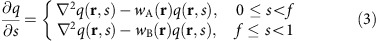


subjected to the initial condition *q*(**r**, 0)=1. As the two ends of the polymer are distinct, a complementary partition function q^*^(**r***, s*) is defined similarly and satisfies the same modified diffusion equation with an initial condition q*(**r**, 1)*=*1. Here, we utilized *s* as a chain contour variable in units of *N*. All lengths were expressed in units of the unperturbed radius-of-gyration of a polymer, *R*_g_=(*Nb*^2^/6)^1/2^, where *b* is the statistical segment length. The solution to the modified diffusion equation was conducted by implementing the pseudo-spectral method^68^. An iterative relaxation of the fields towards their saddle-point values was implemented following ref. 69.

By evaluating *q*(**r***, s*) and its complementary function, the segment volume fractions can be determined as follows


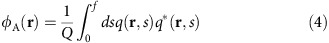



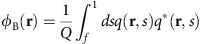


Here we considered a 2D rectangular domain of size (*L*_x_ × *L*_y_=24 × 14R_g_^2^). The SCFT equations were solved on a grid that had an equal lattice spacing of 0.2R_g_ in the *x* and *y* directions. The volume fraction of the polymer *f*_A_ was set to 0.5, whereas the degree of incompatibility *χN* was 17. This resulted in a structure that had the form of stripes (2D projection of in-plane cylinders or out-of-plane lamellae). The sides of the computational cell had periodic boundary conditions simulating an unconfined system in the *x* direction. Confinement was applied to the top and bottom surfaces where both have equal affinity to block *A*.

A masking method was implemented to simulate one-dimensional polymer confinement. A pressure potential *w*_+_=(*w*_B_+*w*_A_)/2 was imposed as a mask on the top and bottom boundaries of the computation domain to simulate the effect of impenetrable walls. The mask had a thickness of four lattice points for every wall with *w*_+_=35. The polymer densities of both *A* and *B* in this region were vanishingly small. On the other hand, a wetting layer that was preferential to *A* was imposed on the inner side of the impenetrable region. It had a thickness of two lattice points. The strength of the exchange potential *w*_−_=(*w*_B_−*w*_A_)/2 in the wetting layer determined the degree of affinity of the surface to *A.* We used a low affinity of *w*_−_=0.5 to simulate weak attraction, whereas a high value of *w*_−_=3 created a uniform wetting layer parallel to the wall.

In the 3D simulation, the computational cell had dimensions of *L*_x_ × *L*_y_ × *L*_z_=11.5 × 11.5 × 4.8R_g_^3^ with number of lattice points (*N*_x_, *N*_y_, *N*_z_)=(48, 48, 40). The base was made square to avoid any bias towards the orientation of the polymer domains. The volume fraction of the polymer *f*_A_ was set to 0.27 and the degree of incompatibility χ*N* was 30. The polymer self-assembled into cylinders with an equilibrium periodicity of ∼3.6R_g_. The computational cell had periodic boundary condition on all side-faces. Again a masking method was used to impose confinement in the vertical direction. A flat top layer mimics the air/polymer (thickness of four lattice points) interface with an inner layer that was attractive to minority polymer *A* (two lattice points). The film thickness was kept constant at 1*L*_0_ (where *L*_0_ is the equilibrium polymer periodicity). On the other hand, the bottom mask consisted of a flat substrate with periodic walls. The walls of the bottom layer were coated with a wetting layer to attract one of the blocks. It had a thickness of two lattice points. The wall thickness is kept constant at three lattice points. Wall spacing and wall height could be varied to study the effect of confinement and commensurability on the polymer orientation. Four trench sizes were studied with trench spacing having values 3*L*_0_, 1.5*L*_0_, 1*L*_0_ and 0.75*L*_0_. These spacings covered the conditions of compression, or tension of polymer chains for incommensurate cases, in addition to commensurate cases. Three different barrier heights were investigated at 0.1*L*_0_, 0.2*L*_0_ and 0.3*L*_0_, however, the change in polymer behaviour was observed between 0.1*L*_0_ and 0.2*L*_0_. Thicker barrier heights only improved confinement.

## Additional information

**How to cite this article:** Tavakkoli K. G., A. *et al*. Multilayer block copolymer meshes by orthogonal self-assembly. *Nat. Commun.* 7:10518 doi: 10.1038/ncomms10518 (2016).

## Supplementary Material

Supplementary Information Supplementary Figures 1-16
