# Supplementary material for: Multilayer block copolymer meshes by orthogonal self-assembly
Source: Nat Commun. 2016 Jan 22;7:10518. doi: 10.1038/ncomms10518 (PMC4736107; doi:10.1038/ncomms10518)
Supplement: Supplementary Information — Supplementary Figures 1-16 [file ncomms10518-s1.pdf]

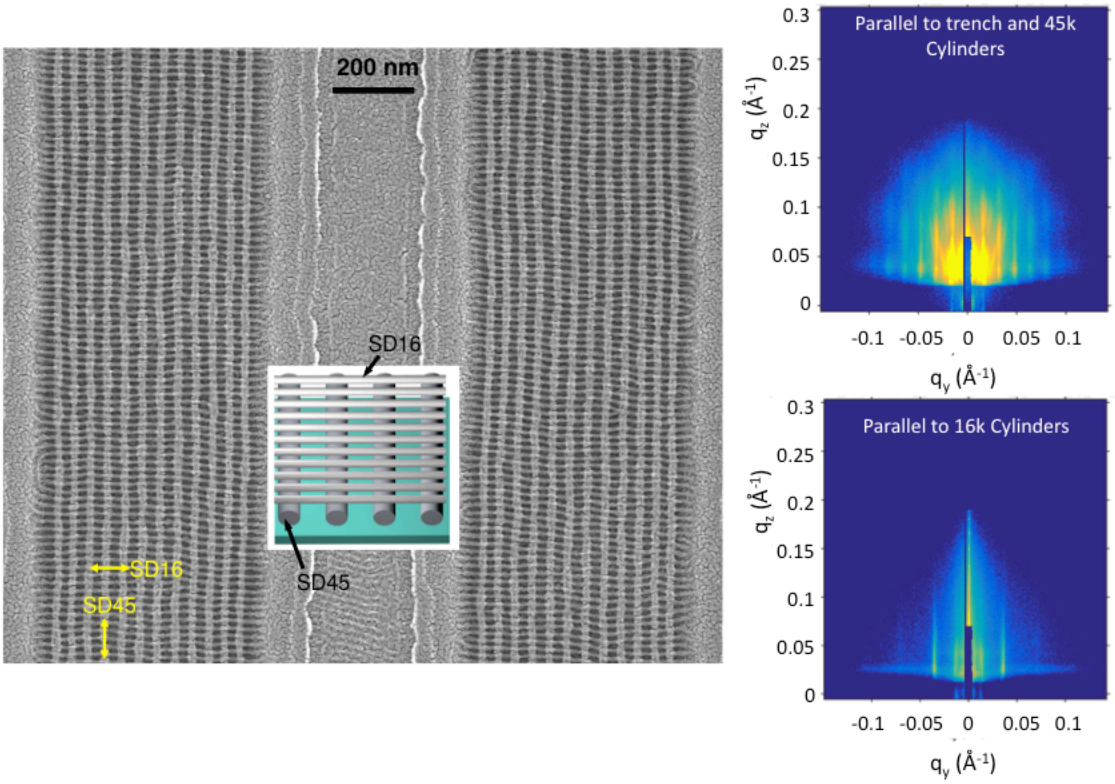

Supplementary Figure 1: Mesh-shaped structure in an interference lithography trench template. Left: The trench template was functionalized with a PDMS brush before spin coating SD45 BCP. The SD45 BCP cylinders (bottom layer) are oriented in the *y*-direction and SD16 BCP cylinders (top layer) were orthogonally self-assembled by the bottom-layer SD45 cylinders to form nanomesh pattern. Right: Example grazing-incidence small-angle x-ray scattering shots, showing large-area orientation correlation. Top Right: GISAXS intensity from beam which was aligned parallel to trench templates and the bottom layer of the aligned SD45 PDMS cylinders. Multiple elongated grating peaks along the q_y direction are indicative of the long-range order of the SD45 cylinders. The diffraction peak spacing is consistent with top-down SEM measurements of SD45 cylinder L_0. Bottom Right: GISAXS intensity from beam which was aligned parallel to the top layer of the aligned SD16 PDMS cylinders. The first order diffraction peak spacing is consistent with top-down SEM measurements of SD16 cylinder L_0. Both of these images were taken at θ (measured critical angle) = 0.2°.

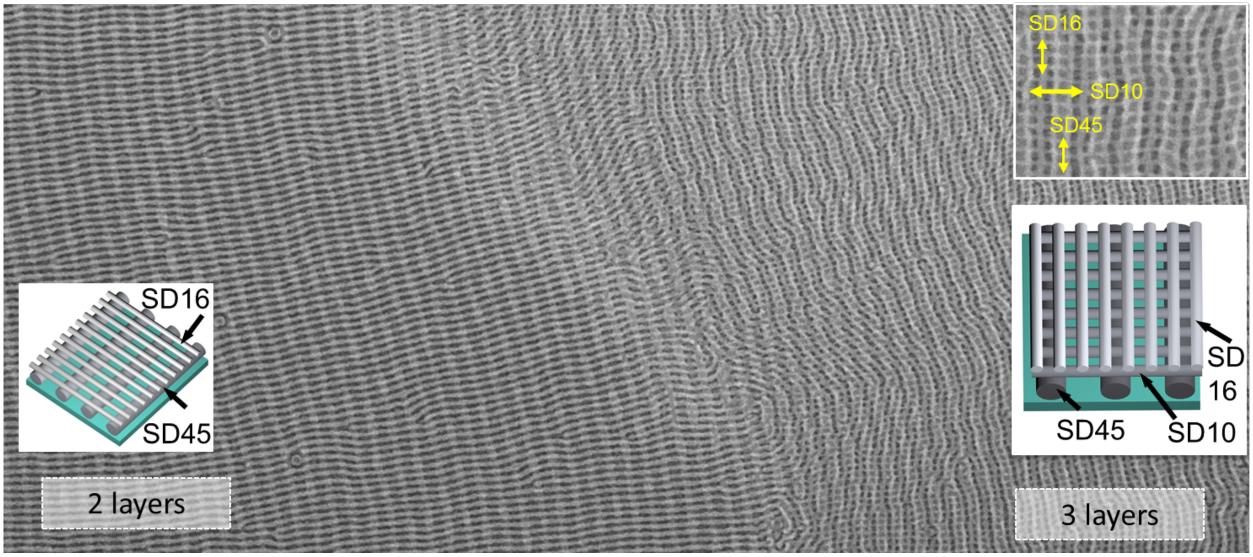

Supplementary Figure 2: SEM Image of multilayer mesh patterns of both two layers (left) and three layers (right). Left: a bilayer of the mesh structure consisting of SD45 BCP on the bottom, and perpendicularly templated SD16 BCP on the top. Right: The image transitions to a mesh with three perpendicularly oriented layers, SD45 BCP on the bottom, SD10 BCP second and SD16 BCP on the top. This transition occurred in an area where the SD10 dewet from the SD45 on the left. Note that in the right of the image the SD16 BCP, as the top layer, is aligned predominantly parallel to the SD45 BCP on the bottom. The inset shows a zoomed-in image of the three-layer mesh, highlighting the different layers. In contrast, the SD16 in the left is orthogonal to the SD45 BCP on the bottom.

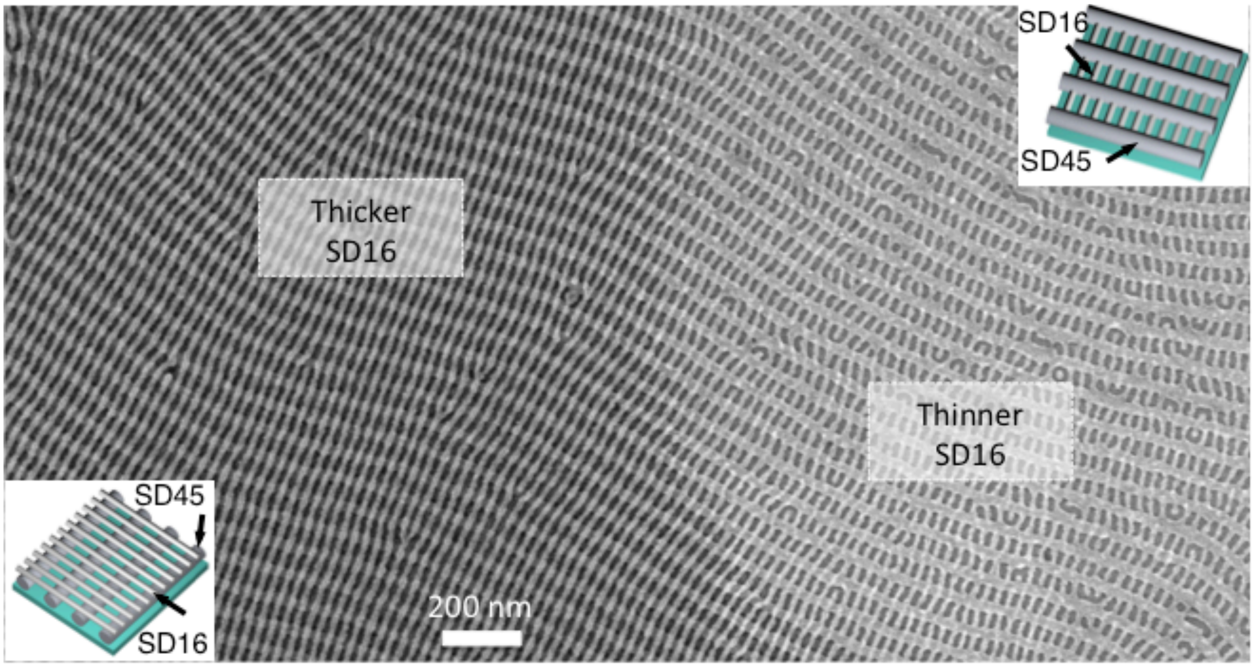

Supplementary Figure 3: SEM image of orthogonal self-assembled nanomesh with varying thickness of second-layer BCP. When the SD16 BCP is thicker (left), the mesh forms in a bilayer, and the SD16 cylinders were continuous over the top of SD45 bottom-layer lines. When the second-layer SD16 BCP is thinner than a monolayer (right), the SD16 cylinders spanned between the bottom SD45 cylinders.

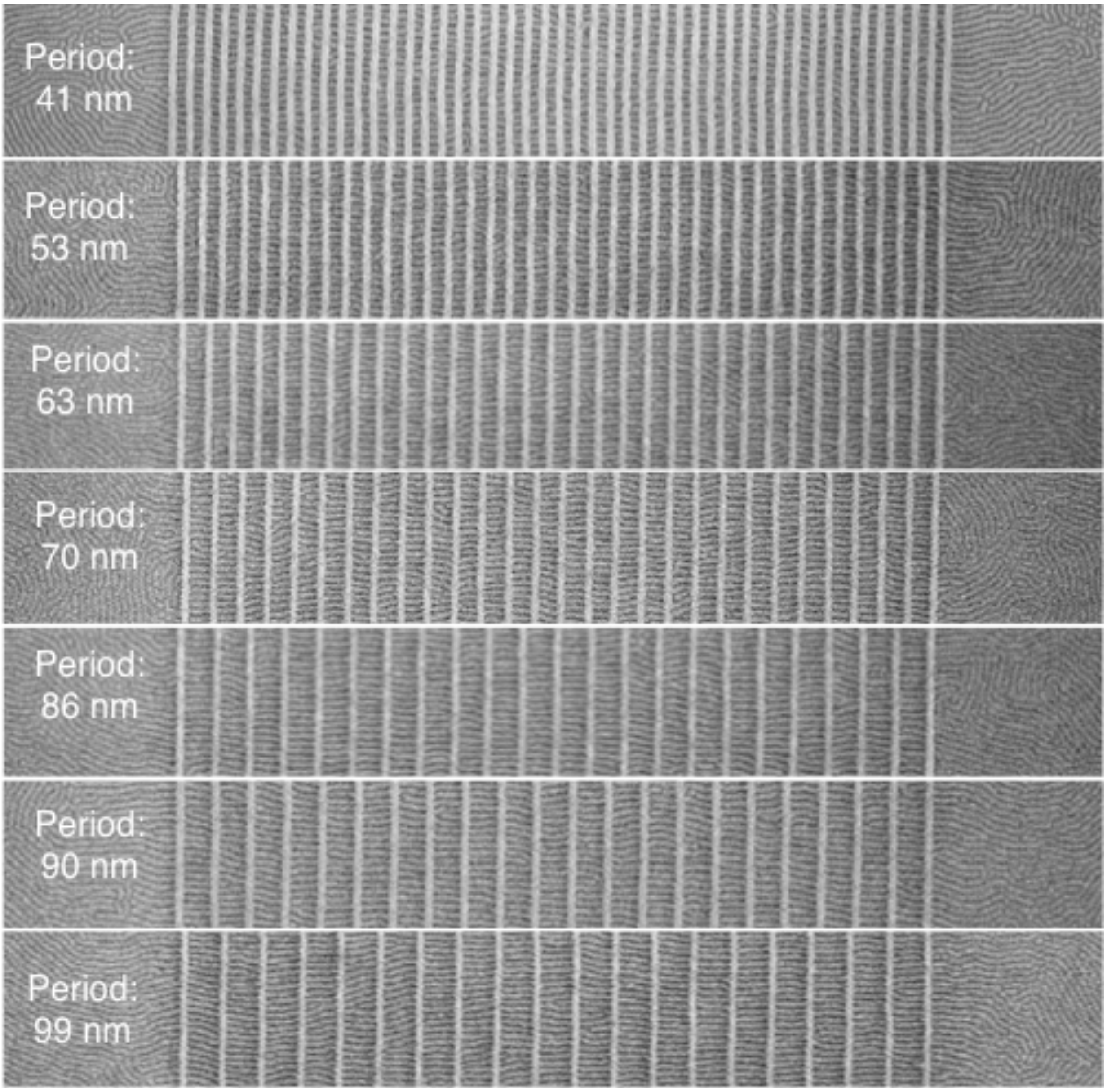

Supplementary Figure 4: SEM images of orthogonal alignment of SD16 BCP over HSQ line templates at varying periods. Within an HSQ line array of varying periods, SD16 BCP cylinders oriented orthogonal to the template patterns. This effect was observed for a large range of periods and the shown images are only selected examples. Orthogonal self-assembly was found to be independent of template period at these length scales.

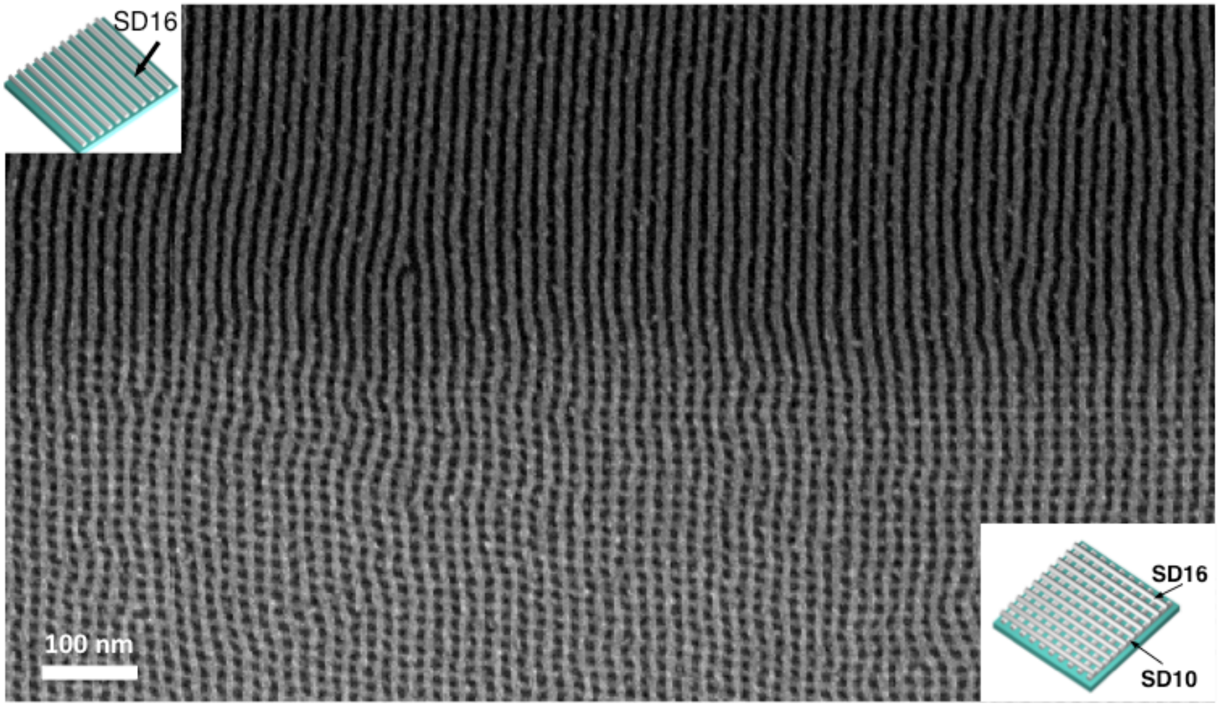

Supplementary Figure 5: SEM image of ultra high-density nanomesh patterns of low molecular weight BCP bilayers. Top, only a monolayer of SD16 formed a grating. Bottom, the nanomesh formed via orthogonal self-assembly of SD16 BCP on top of SD10 BCP.

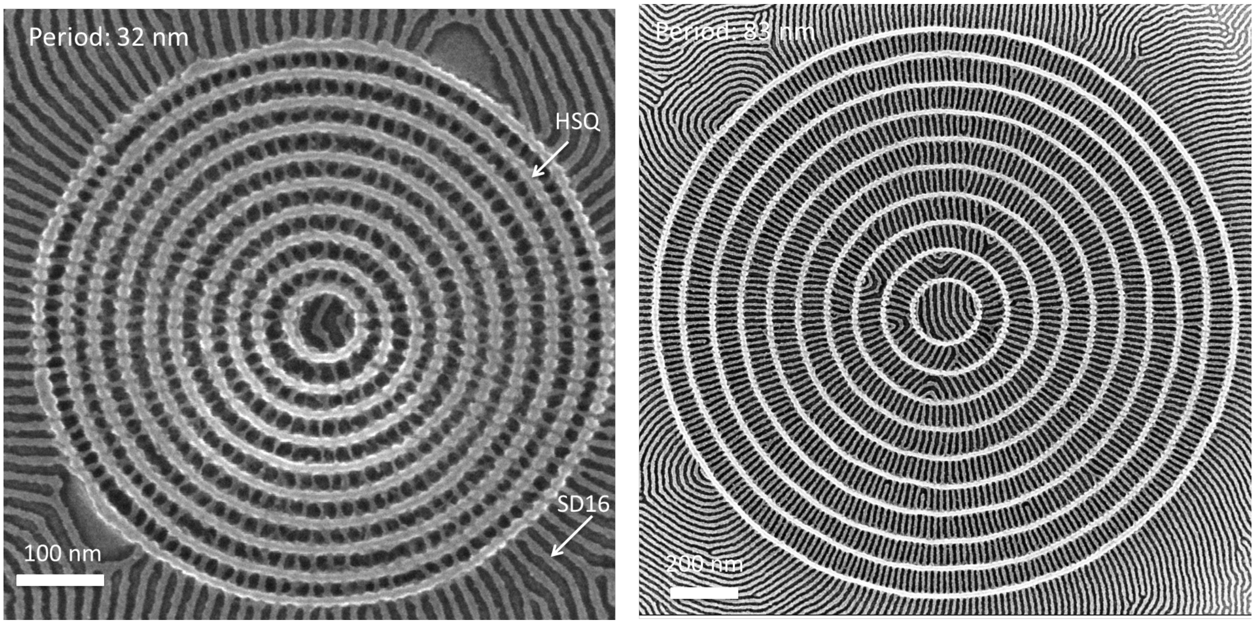

Supplementary Figure 6: SEM images of orthogonal alignment of SD16 BCP over concentric EBL-patterned HSQ circles. SD16 cylinders aligned radially to form wagon wheel patterns. This orthogonal self-assembly of wagon-wheels was observed for varying periods of concentric HSQ circles. As two examples, the periods of the templating circles shown are 32 nm (left) and 83 nm (right).

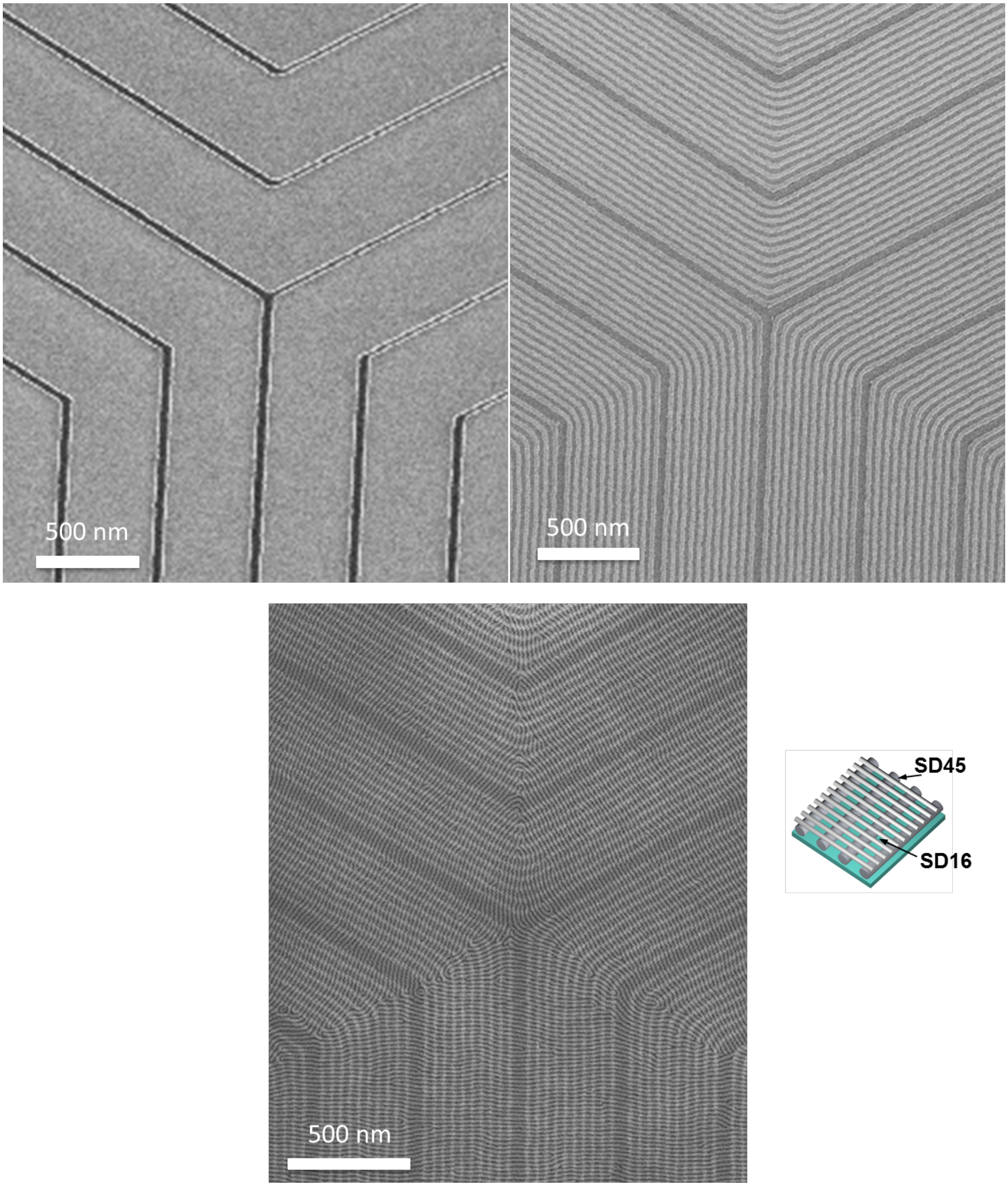

Supplementary Figure 7: SEM images of nanomesh patterns oriented in a Y-shape. Top left: SEM image of the PMMA EBL template fabricated in a Y-shape. Top right: SEM image of SD45 cylinders parallel to the PMMA Y-shaped template. After O_2 RIE, the PMMA template was removed along with the PS block. Bottom: SEM image of the nanomesh Y-shaped pattern in which the SD16 BCP was directed orthogonally to the underlying SD45 BCP. Note that the SD16 BCP crossed over the gaps left by the PMMA template.

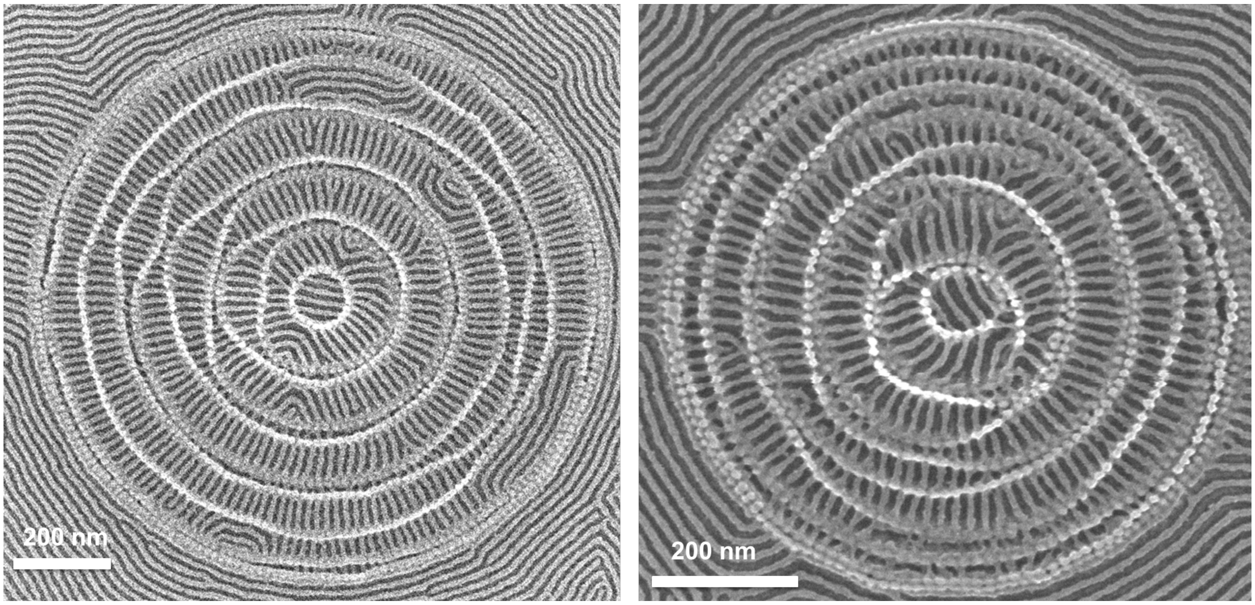

Supplementary Figure 8: SEM images of orthogonal alignment of SD16 BCP over imperfect concentric HSQ circles. Orthogonal alignment was observed even though the period between templating circles was inconsistent. In these cases, the HSQ circles had toppled over both inwards and outwards during the development of the HSQ structures.

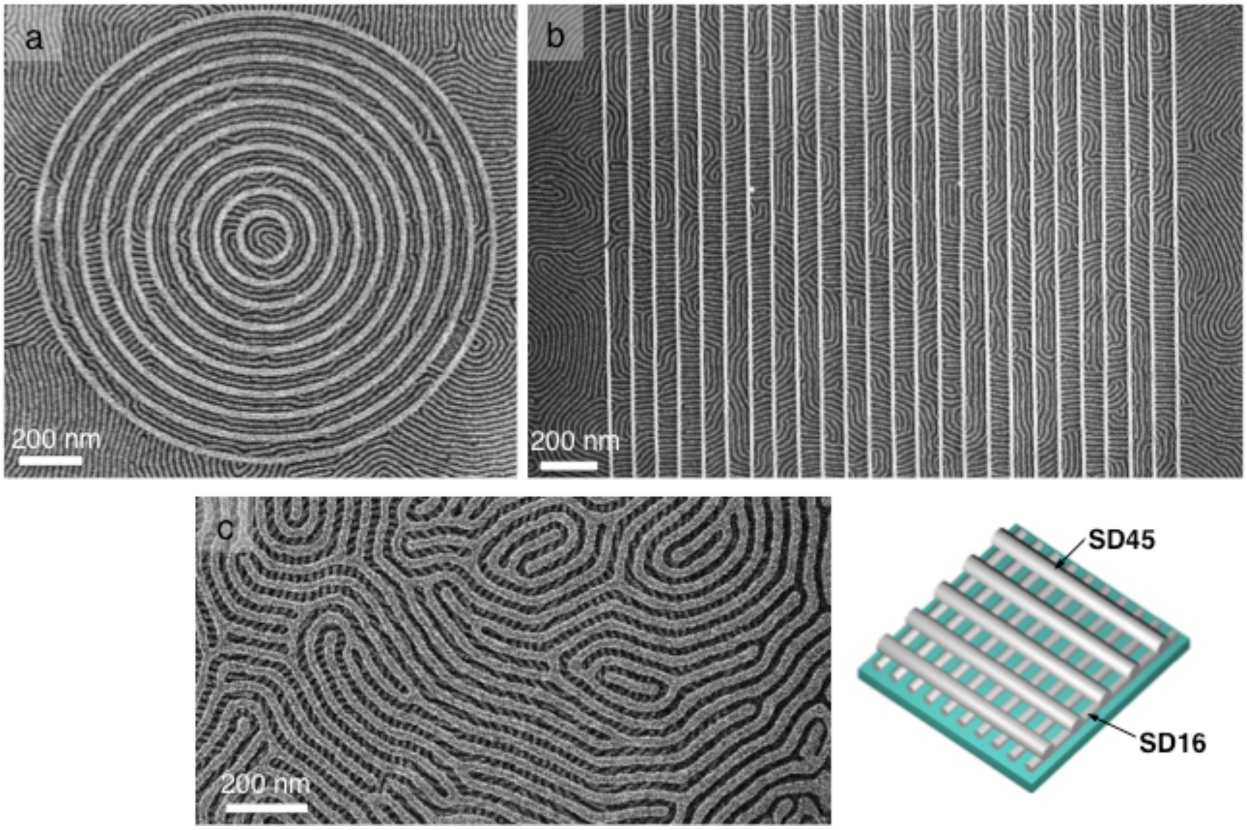

Supplementary Figure 9: Imperfect orthogonal alignments in thin bottom-layer topography. SEM images of various imperfect orthogonal alignments in which the bottom-layer topography is too thin to induce complete orthogonal orientation of the top-layer. Bottom-layer templates include a) HSQ (1%) concentric circles with an SD16 BCP top-layer, b) HSQ (1% solution) line arrays with an SD16 BCP top-layer, and c) SD16 BCP Cylinders with an SD45 BCP top-layer.

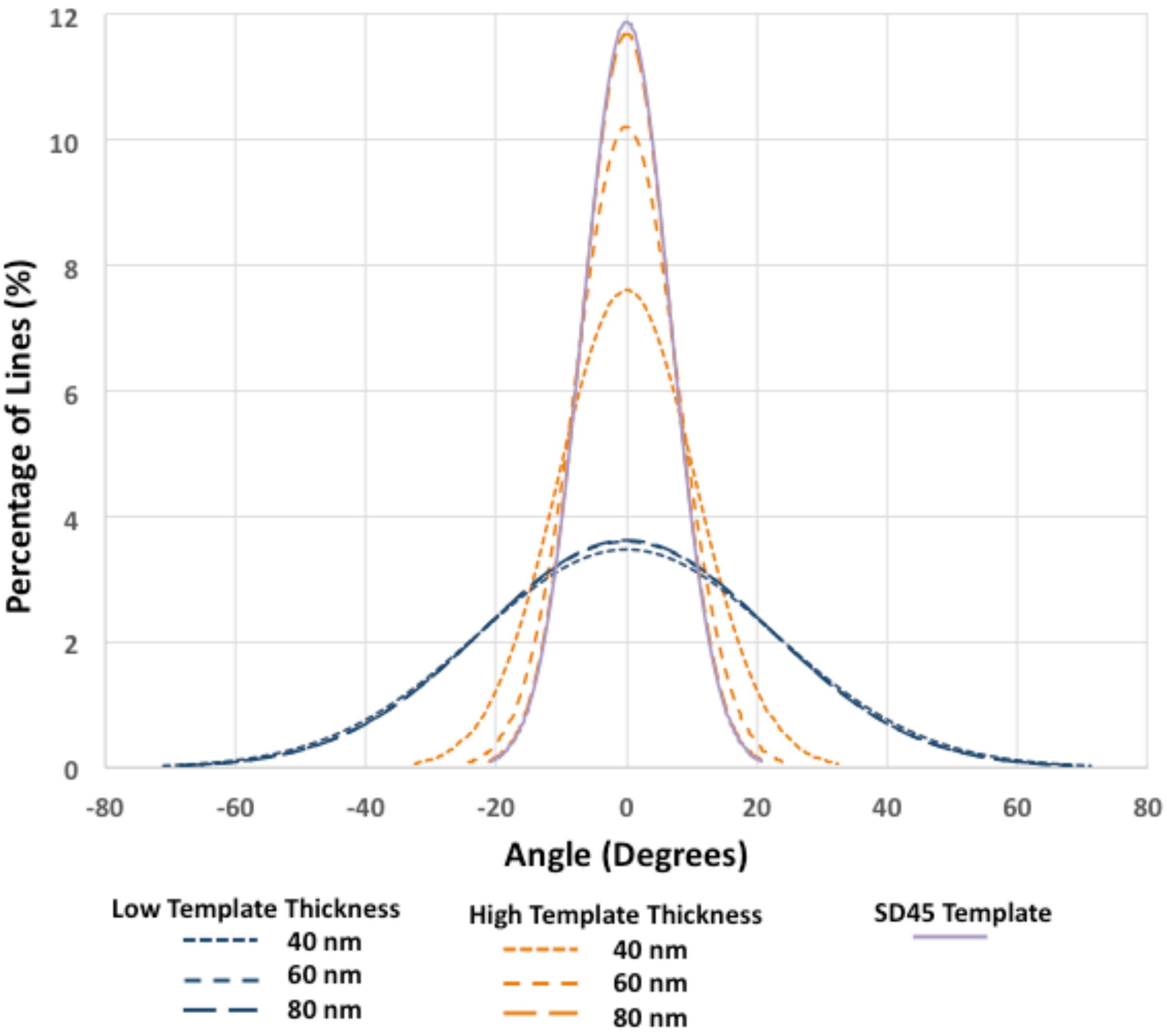

Supplementary Figure 10: Plots of the distribution of SD16 cylinder angles. SEMs were analysed to extract the orientation angles of SD16 cylinders on top of different substrates and the normal fit is shown. Templates of HSQ line arrays with both thick (estimated 50 nm as-spun, as in Supplementary Fig. 4) and thin (estimated 15 nm as-spun, as in Supplementary Fig. 9), as well as SD45 cylinder, were analyzed. The pitches for the HSQ lines were 40, 60, and 80 nms. The impact of the template thickness is evident, as orientation angles have a higher deviation for thinner templates than for thicker templates. Furthermore, the BCP-on-BCP mesh, with the SD45 template, produced the sharpest distribution because the SD16 cylinders spanned the bottom template and therefore the angle was more consistent from template-line to template-line. It should be noted that this analysis is not meant to be representative of the fundamental orientation characteristics of the Orthogonal Self-Assembly Method, but as a representation of the impact of the height of the template on the distribution of orientations.

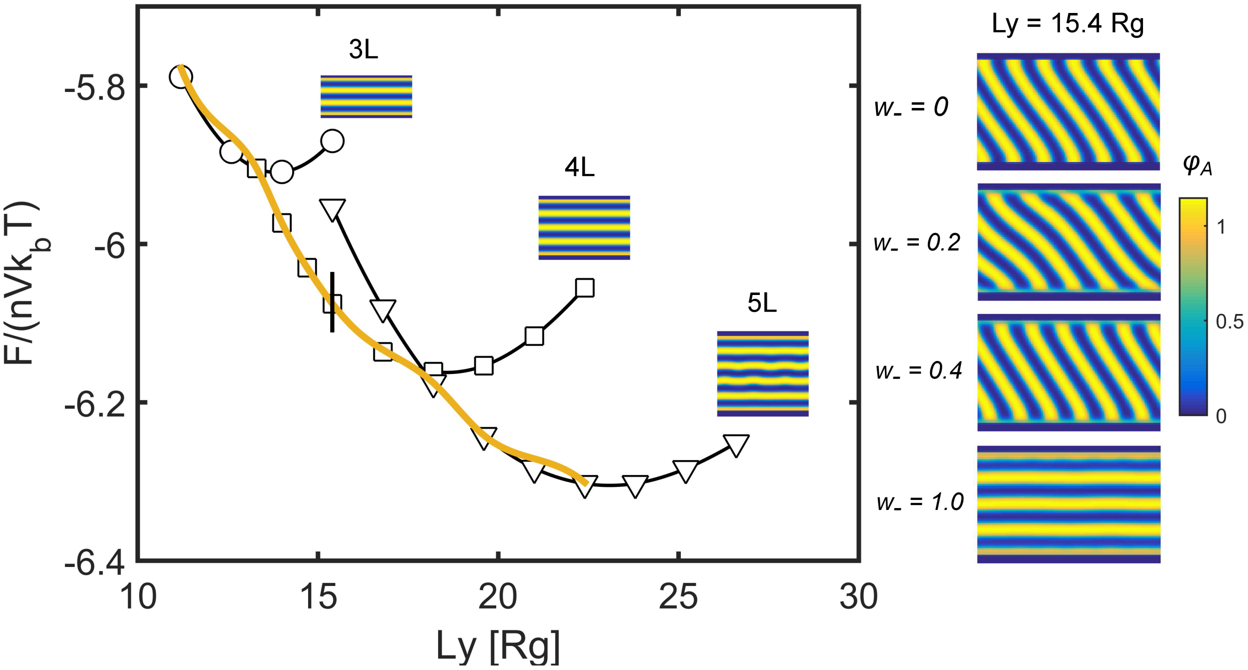

Supplementary Figure 11: Free energy analysis for multiple polymer domains under the confinement as a function of wall separation. Free energy was calculated by seeding the simulation with the desired structure. SCFT simulation from random fields (thick continuous line) indicates the equilibrium structure. Equilibrium structure of cell height of $L_y = 15.4R_g$ at varying surface attraction strength is shown on the right. Orthogonal structures prevailed for small attractive fields ($w_- < 0.4$). The larger attraction strength ($w_- = 1.0$) resulted in a parallel structure. As was discussed, the orthogonal structure persisted for small wall attraction strength. In order to ensure that the orthogonal structure was not caused by incommensurability between domain periodicity and wall separation, free energy calculations for multiple parallel structures were conducted. Cell height of $L_y = 15.4R_g$ was commensurate with the polymer periodicity as shown in this figure. For neutral walls ($w_- = 0$), an orthogonal structure of the microdomains prevailed. The structure persisted until ($w_- = 0.4$), after which the polymer made the transition to a parallel structure.

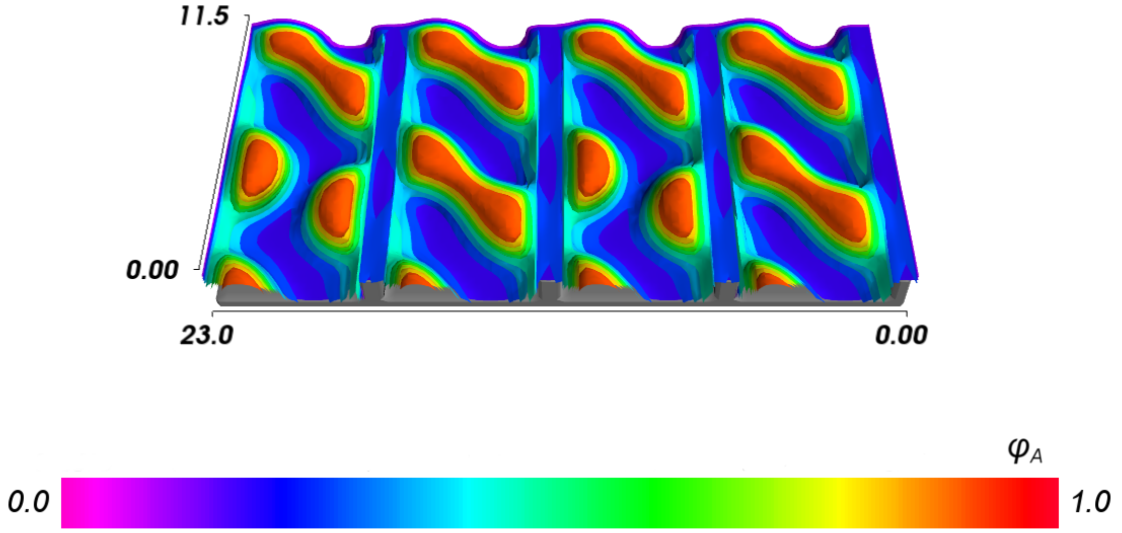

Supplementary Figure 12: Isosurfaces for *A* density of a pre-orthogonal structure. An intermediate state of polymer self-assembly showing that polymer microdomains starting from walls merge to create an orthogonal structure. Where microdomains merged, a neck was formed with a smaller radius in agreement with the experiments in Supplementary Figs 4, 5 and 6. Top wetting layer in 3D simulations was removed to reveal the structure. Computational cell was doubled to demonstrate trench periodicity. Dark gray substrate was added for clarity.

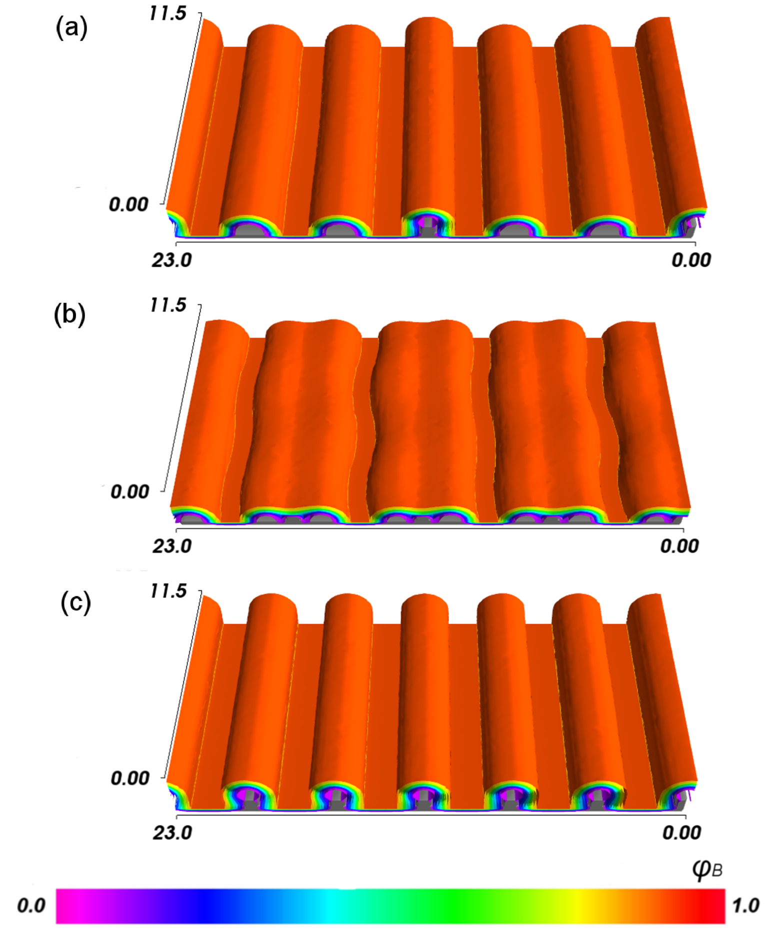

Supplementary Figure 13: Isosurfaces for *B* density when the template is strongly attractive to minority block (*A*). The parallel structure was generated by increasing the attraction of topography. Top wetting layer in 3D simulations was removed to reveal structure. Computational cell was doubled to demonstrate trench periodicity. Dark gray substrate was added for clarity.

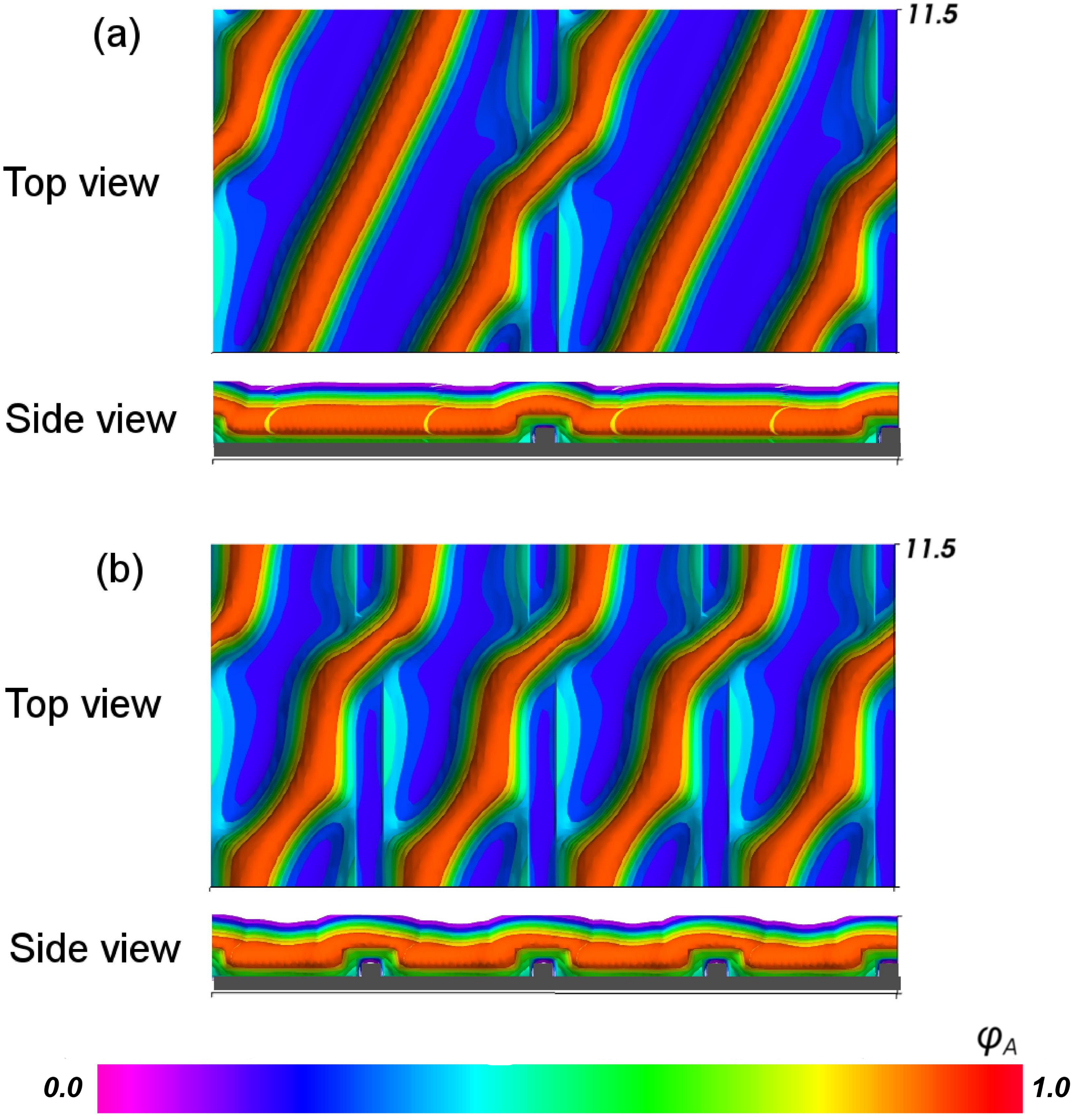

Supplementary Figure 14: Isosurfaces for *A* density when the template is attractive to the minority block (*A*). For low surface attraction to *A*, and low wall height (<10% of film thickness), microdomains on both sides of walls merge allowing the microdomain to span multiple trenches. Top wetting layer in 3D simulations was removed to reveal structure. Computational cell was doubled to demonstrate trench periodicity. Dark gray substrate was added for clarity.

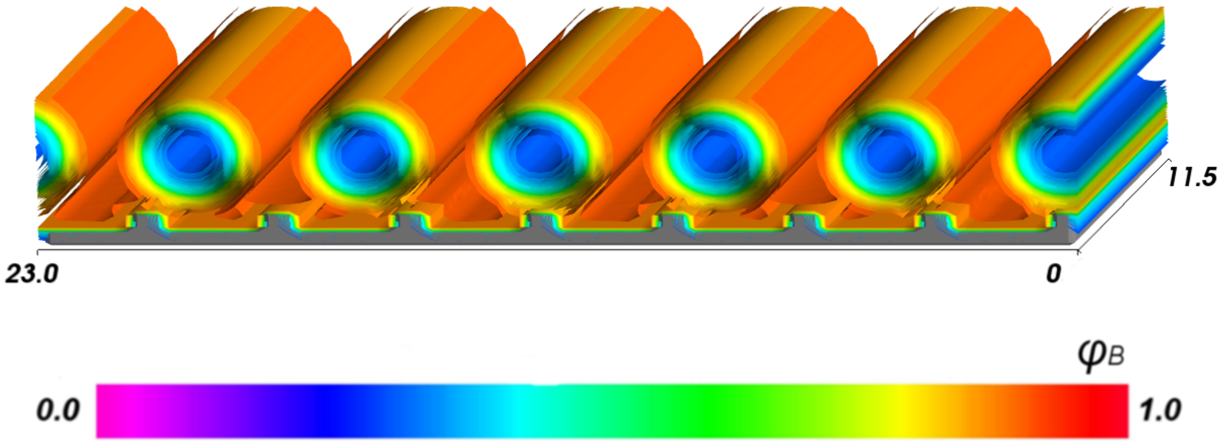

Supplementary Figure 15: Isosurfaces for *B* density at small wall height (< 10% film thickness). Cylinders maintained their natural periodicity (six cylinders) independent of trench spacing ($0.75L_0$) because the cylinders could lie above the barriers. Top wetting layer in 3D simulations was removed to reveal structure. Computational cell was doubled to demonstrate trench periodicity.

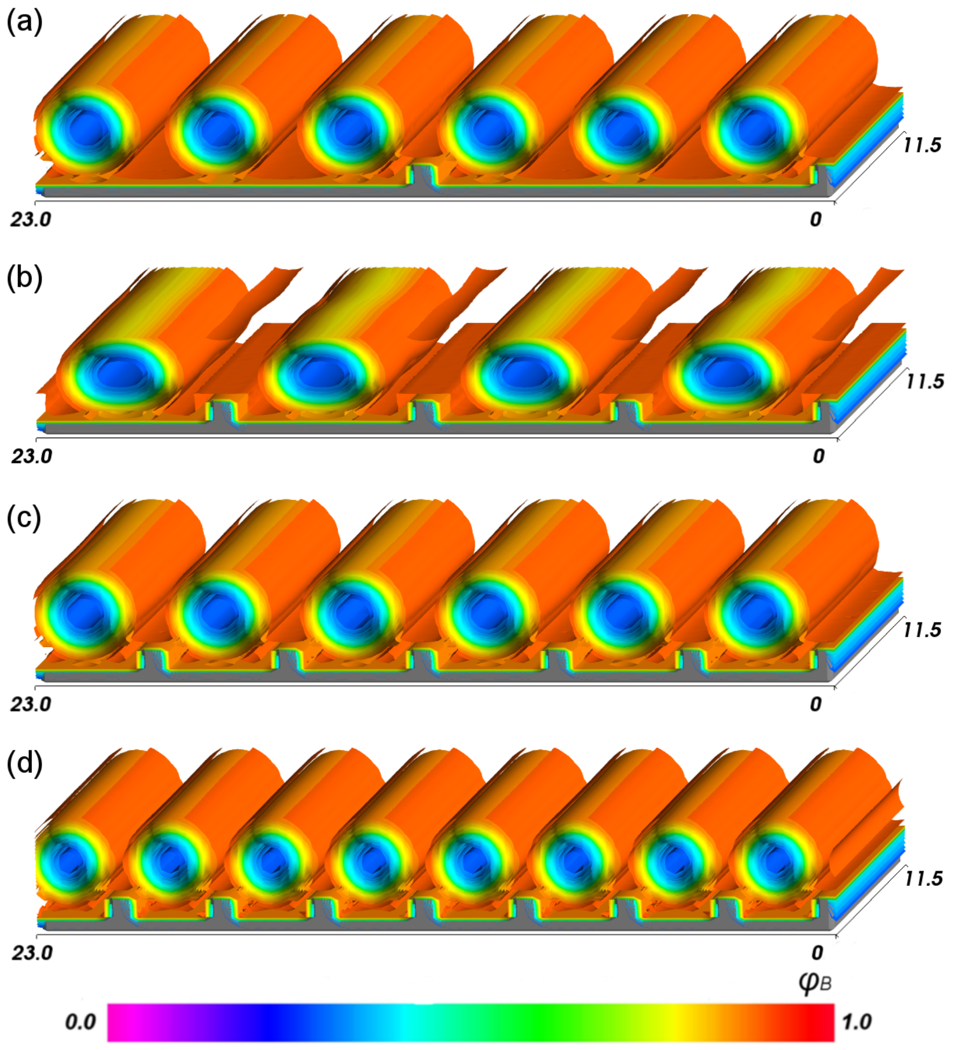

Supplementary Figure 16: Isosurfaces for *B* density when the template is attractive to the majority block (*B*). A parallel structure was generated when the surface was attractive to *B*. With wall height of > 10% of film thickness, cylinders were confined inside trenches causing severe strain when trench spacing was incommensurate with polymer periodicity. Top wetting layer in 3D simulations was removed to reveal structure. Computational cell was doubled to demonstrate trench periodicity.
